# The Molecular Chaperone Hsp90α Is Required for Meiotic Progression of Spermatocytes beyond Pachytene in the Mouse

**DOI:** 10.1371/journal.pone.0015770

**Published:** 2010-12-31

**Authors:** Iwona Grad, Christopher R. Cederroth, Joël Walicki, Corinne Grey, Sofia Barluenga, Nicolas Winssinger, Bernard De Massy, Serge Nef, Didier Picard

**Affiliations:** 1 Département de Biologie Cellulaire, Université de Genève, Sciences III, Genève, Switzerland; 2 Département de Médecine Génétique et Développement, Université de Genève, Centre Médical Universitaire, Genève, Switzerland; 3 Institut de Génétique Humaine, IGH – CNRS, Montpellier, France; 4 Institut de Science et d'Ingénierie Supramoléculaires, Université de Strasbourg, Strasbourg, France; Fred Hutchinson Cancer Research Center, United States of America

## Abstract

The molecular chaperone Hsp90 has been found to be essential for viability in all tested eukaryotes, from the budding yeast to Drosophila. In mammals, two genes encode the two highly similar and functionally largely redundant isoforms Hsp90α and Hsp90β. Although they are co-expressed in most if not all cells, their relative levels vary between tissues and during development. Since mouse embryos lacking Hsp90β die at implantation, and despite the fact that Hsp90 inhibitors being tested as anti-cancer agents are relatively well tolerated, the organismic functions of Hsp90 in mammals remain largely unknown. We have generated mouse lines carrying gene trap insertions in the *Hsp90α* gene to investigate the global functions of this isoform. Surprisingly, mice without Hsp90α are apparently normal, with one major exception. Mutant male mice, whose Hsp90β levels are unchanged, are sterile because of a complete failure to produce sperm. While the development of the male reproductive system appears to be normal, spermatogenesis arrests specifically at the pachytene stage of meiosis I. Over time, the number of spermatocytes and the levels of the meiotic regulators and Hsp90 interactors Hsp70-2, NASP and Cdc2 are reduced. We speculate that Hsp90α may be required to maintain and to activate these regulators and/or to disassemble the synaptonemal complex that holds homologous chromosomes together. The link between fertility and Hsp90 is further supported by our finding that an Hsp90 inhibitor that can cross the blood-testis barrier can partially phenocopy the genetic defects.

## Introduction

Hsp90 is an ubiquitous, highly conserved protein, comprising up to 2% of total cell proteins even under non-stressed conditions. It facilitates the folding and activity of a large number and variety of client proteins. The list of Hsp90 interacting proteins has already grown to almost 300 proteins and it keeps growing. The activity of Hsp90 is modulated by the interaction with a variety of co-chaperones, which can act as regulators of the ATPase activity, influence the choice of clients, target client proteins for degradation, recruit other co-chaperones, or affect the cellular localization of the clients [1]–[4] (for a comprehensive and updated summary of Hsp90 facts, see http://www.picard.ch/downloads/Hsp90facts.pdf]). In humans and mice, there are two cytosolic Hsp90 isoforms, encoded by two separate genes, Hsp90α (gene *Hsp90aa1*) and Hsp90β (gene *Hsp90ab1*). With 85.8% sequence identity and 93.4% similarity, the two isoforms are highly homologous. Whereas Hsp90β is more or less constitutively and ubiquitously expressed, the expression of Hsp90α is heat-inducible and more tissue-specific [5].

So far, there is only limited evidence for isoform-specific functions. Provided the two isoforms are expressed at all, they are thought to be largely redundant. The known exceptions include the specific participation of Hsp90α in antigen processing [6] and in blocking caspase-2 activation [7]. It has also been demonstrated that Hsp90α can be secreted to promote the maturation of matrix metalloprotease 2 and cell invasiveness in metastasis [8], and cell migration in wound healing [9]. Recently, it was suggested that Hsp90α might play a role in female meiosis in the mouse, notably in the G2/M transition [10]. A role for Hsp90 in spermatogenesis had first been described in *Drosophila melanogaster*, where males with certain transheterozygous combinations of mutant *hsp90* alleles are sterile and display a disrupted meiosis, possibly due to a defect in microtubule dynamics [11]. A study on testis in newt showed a role for Hsp90β in prolactin-induced apoptosis of spermatogonia [12].

The *in vivo* role of the Hsp90 machinery has been mainly investigated by genetic studies in yeast and pharmacologically with mammalian tissue culture cells. In contrast, the genetic analysis of the Hsp90 chaperone machine in the mouse is still in its infancy. Although an Hsp90β gene disruption was found to be early embryonic lethal ten years ago [13], this finding was not further investigated. Mutational analyses of some of the co-chaperones such as FKBP51, FKBP52 and p23 have highlighted the complexity of the Hsp90 machinery and the fact that interesting insights can be gained from genetically ablating components of this important housekeeping chaperone machine [14]–[16]. The aim of this study was to investigate genetically the *in vivo* role of Hsp90α, the other core component of this molecular machine, in the mouse.

## Results

### Generation of Hsp90α gene disruption mutants in the mouse

**Figure 1:**
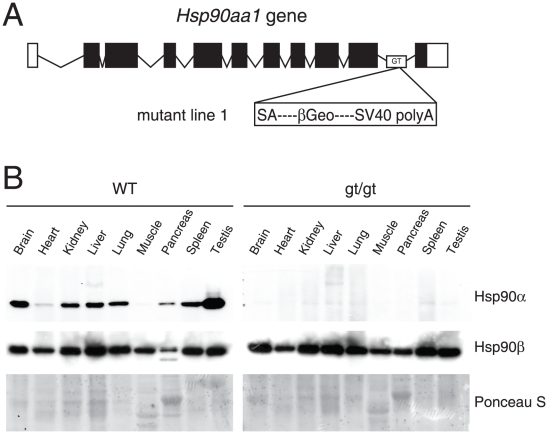
Functional disruption of the Hsp90α gene in the mouse. (A) Schematic representation of the disruption of the mouse *Hsp90aa1* gene by insertion of a gene trap (GT) in intron 10 (in mutant mouse line 1). Open and black boxes indicate non-coding and coding exons, respectively. The gene trap consists of a splice acceptor (SA), a β-galactosidase-neomycin resistance fusion (βGeo) and an SV40 polyadenylation site. (B) Immunoblots showing the expression of Hsp90α and Hsp90β in a panel of tissues in wild-type (WT) and mutant (gt/gt) mice (of 136 days postnatal). The Ponceau S staining of the immunoblot filter gives an indication of protein loading.

In order to assess the role of Hsp90α *in vivo*, a mutant mouse line was established from an embryonal stem cell clone carrying a gene trap insertion in the last intron of the *Hsp90aa1* gene (Fig. 1A). This particular insertion of a gene trap vector into intron 10 could potentially encode an in-frame fusion protein consisting of a truncated Hsp90α lacking the C-terminal 36 amino acids and a β-galactosidase-neomycine resistance gene (βGeo). The very C-terminal domain of Hsp90α comprises the conserved sequence motif MEEVD, which is essential for the interaction with the tetratricopeptide repeats present in some Hsp90 co-chaperones. This is notably the case for the large immunophilins Cyp-40, FKBP51 and FKBP52, the serine–threonine protein phosphatase 5 and CHIP [17], [18]. A more extensive portion of the C-terminal domain is required for dimerization and for viability in the budding yeast [19]. Whereas a truncation mutant of the C-terminal 24 amino acids of yeast Hsp90 is sufficient for viability, a truncation of 57 amino acids is not [20]. Moreover, while a wild-type version of mammalian Hsp90α complements the budding yeast [19], [21], a Hsp90αΔ35 mutant, which can be considered intermediate between the two afore-mentioned yeast mutants, is severely defective for complementation (Morag McLean and DP, unpublished results). Therefore, it is likely that such a truncation, even if it were expressed at normal levels, would be largely dysfunctional.

An immunoblotting experiment with specific antibodies showed that Hsp90α is expressed in a pronounced tissue-specific pattern with high levels in testis and almost none in heart and skeletal muscle (Fig. 1B). By comparison, Hsp90β is expressed much more evenly across the same panel of tissues. To determine if any residual or fused Hsp90α protein persists in gene disruption mutants, we analysed the same tissues from homozygous mutant mice. Even though the polyconal antiserum against Hsp90α that was used would have been able to recognize the slightly truncated form, no wild-type Hsp90α or Hsp90αΔ36-βGeo could be detected in any of the analysed tissues (Fig. 1B, and data not shown) indicating that the gene disruption leads to a loss of all or almost all Hsp90α protein. It is noteworthy that the absence of Hsp90α is not compensated by an increase in Hsp90β. In view of subsequently confirming the key results obtained with this particular gene disruption line, we also generated and investigated another *Hsp90aa1* gene trap insertion mutant (gene trap mutant 2), in which the gene trap insertion occurred before the first coding exon. We expected this insertion to result in a complete disruption of the Hsp90α transcript and of Hsp90α protein expression (Fig. S1; see below). Since the testicular phenotype obtained for both lines appeared to be identical (Fig. S1), we concluded that both lines correspond to functional gene disruptions of the *Hsp90aa1* gene, and continued our analysis primarily with the gene trap mutant 1.

### Hsp90α is required for spermatogenesis

**Figure 2:**
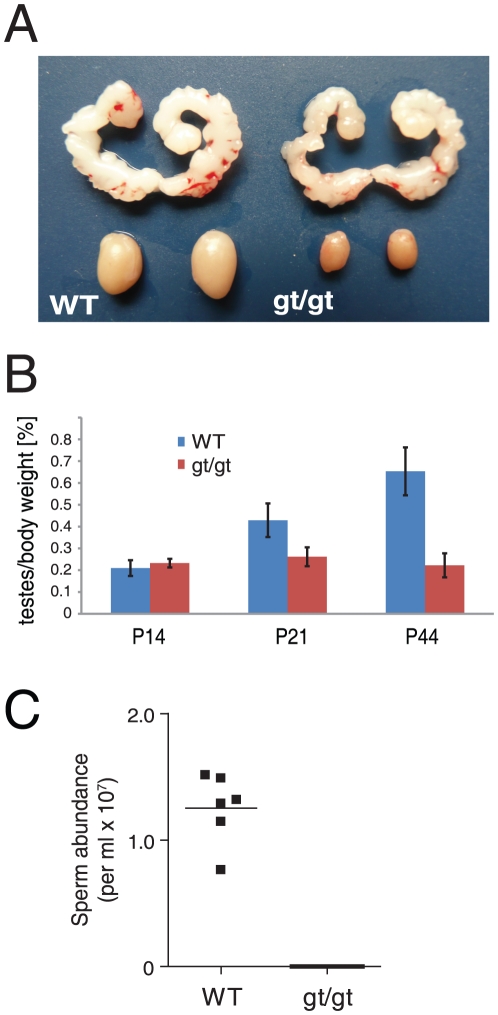
Absence of Hsp90α leads to atrophic testis and azoospermia. (A) Morphology of seminal vesicles and testis of wild-type (WT) and *hsp90α^gt/gt^* mutant (gt/gt) mice at P44. (B) Time course analysis of testis weight in WT and mutant (gt/gt) animals. n≥4. (C) No mature sperm cells could be detected in the epididymis of one year old *hsp90α^gt/g^* ^t^ animals.

Male and female mice homozygous for the gene trap mutation 1, from here on referred to as *hsp90α^gt/gt^*, were viable, grew to adulthood normally and appeared to have normal sexual behaviour when compared to control littermates. Female *hsp90α^gt/gt^* mice were fertile and displayed no obvious ovarian abnormalities (data not shown). In *hsp90α^gt/gt^* males, testicular descent occurred and internal reproductive organs such as seminal vesicles and prostate were normally masculinized (Fig. 2A, and data not shown). Male *hsp90α^gt/gt^* mice exhibited normal androgen-dependent behaviour including mounting and copulatory activity (data not shown). However, they appeared to be sterile as repeated breeding between *hsp90α^gt/gt^* males and wild-type females failed to produce any offspring. When the testicular weight of *hsp90α^gt/gt^* males from postnatal day 14 (P14) until P44 was analysed, it showed a progressive reduction in size, reaching 66% at postnatal day P44 compared to that of wild-type littermates (Fig. 2B). A sperm count analysis revealed that no spermatozoa were present in the caudal epididymis of one year old *hsp90α^gt/gt^* males (Fig. 2C). The same was true for 9 months old males whereas normal sperm counts were found in *hsp90α^+/gt^* heterozygous littermates (data not shown). Thus, except for azoospermia (“absence of sperm”), it appeared that Hsp90α function is dispensable for development and basic physiology.

### Hsp90α is important for meiotic progression in testis

**Figure 3:**
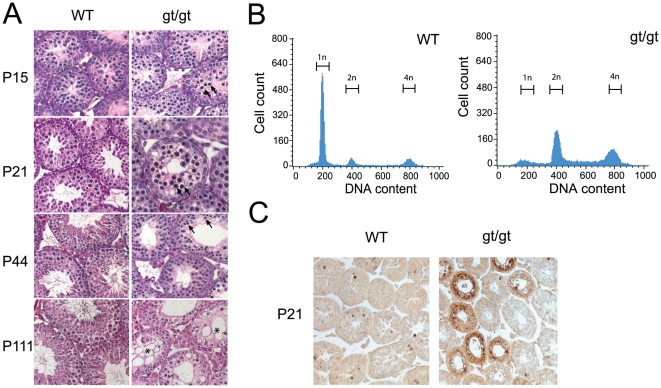
Meiotic arrest and apoptosis in *hsp90α^gt/gt^* mutant testis. (A) Histological sections of testes of of wild-type (WT) and mutant (gt/gt) mice at 15, 21, 44 and 111 days after birth. In the righthand panels, arrows point out apoptotic nuclei and the asterisk in the P111 section indicates degenerating tubules. (B) Flow cytometric analysis of the DNA contents of the whole testis cell population at P44. (C) TUNEL analysis of sections of P21 testes.

To determine where and when the defect in testis development begins in *hsp90α^gt/gt^* males, we compared the development of mutant and control testes from P0 to P44, when the first spermatogenic wave is completed, by histology (Fig. 3A). Prior to P15, mutant and control testes appeared morphologically indistinguishable, indicating that immature Sertoli cells, spermatogonia and early meiotic germ cells developed normally (data not shown). The first abnormalities began to appear at P15 when *hsp90α^gt/gt^* mutant testes ceased to grow (Fig. 3A). The detailed histological examination of P21, P44 and P111 *hsp90α^gt/gt^* mutant testes revealed that meiosis is impaired. It does not progress further than spermatocyte differentiation as no post-meiotic spermatids were observed, and mature spermatozoa in the seminiferous tubules and epididymal ducts were completely missing. Already at P15, when germ cells reach the pachytene stage, cells with typical apoptotic morphology were present in a few tubules. Later at P21 and P44, the phenotype became more severe with a variety of defects ranging from the presence of vacuoles in some mutant tubules lacking spermatocytes to a Sertoli-cell-only (SCO) phenotype at P111 in some cases (Fig. 3A). A lot of pycnotic and multinuclear cells, and primary spermatocytes could be observed suggesting an arrest at the pachytene stage of meiosis I. To confirm the absence of post-meiotic cells, we analysed testicular cell populations at P44 by flow cytometry. The experiment confirmed the absence of 1n cells in the testis of *hsp90α^gt/gt^* mice, normally present at this stage of spermatogenesis as shown for wild-type littermates (Fig. 3B). Moreover, the flow cytometric analysis revealed an increased 4n cell population suggesting a meiotic arrest at prophase I.

Since *hsp90α^gt/gt^* seminiferous epithelium lacked post-meiotic cells, we used a TUNEL assay to investigate whether the missing cells are eliminated by apoptosis. *hsp90α^gt/gt^*murine testes at P21 contain significantly more apoptotic cells than those of wild-type controls (Fig. 3C). Thus, the loss of Hsp90α function results in male-specific infertility due to an incapacity of germ cells to complete meiosis leading to a spermatogenesis arrest, and ultimately cell death of meiotic spermatocytes.

### The testicular phenotype is restricted to germ cells

**Figure 4:**
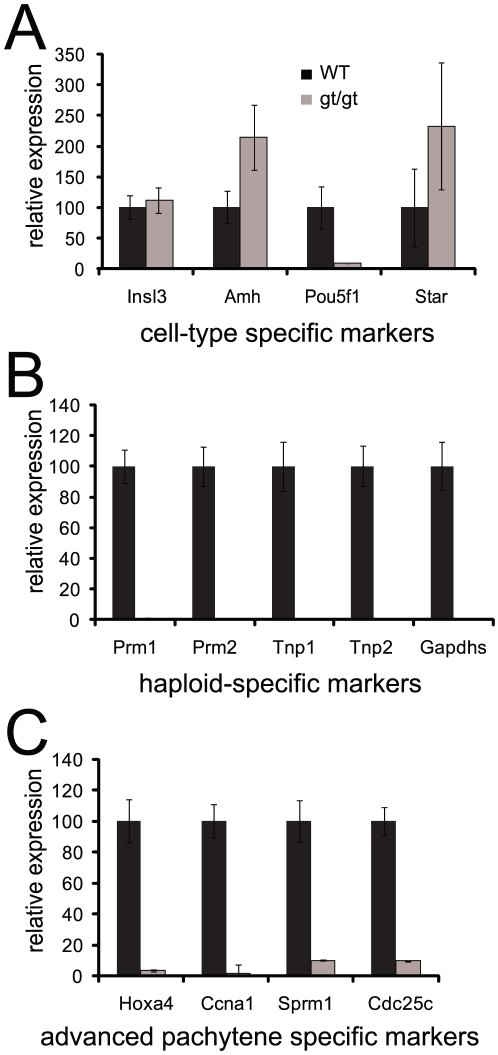
Quantitative RT-PCR analysis of specific markers indicates pachytene arrest in the germ cells of *hsp90α* ^gt/gt^ mutant mouse testis. RNA samples are from P44 mice. (A) Markers of testis compartments. Insl3 and Star, Amh, and Pou5f1 are for Leydig, Sertoli, and germ cells, respectively. (B) Complete absence of post-meiotic markers. (C) Markers of advanced stages of meiosis.

To investigate which testicular cell type might be affected in the resulting phenotype, we measured the expression of a set of markers at P44 to assess the relative proportions of Leydig cells, Sertoli cells, and germ cells. The quantitative PCR data showed that there are no major differences in expression of specific markers for the somatic Leydig (Insl3 and Star) and Sertoli (Amh) cells between *hsp90α* mutant and wild-type testes while the germ cell marker Pou5f1 (Oct4) is practically absent in the mutant (Fig. 4A). Sertoli cell dysfunction can be associated with infertility. However, as pointed out above, *hsp90α^gt/gt^* mutant mice had seminal vesicles of normal size (Fig. 2A), and the maturation of Sertoli cells appeared normal, as reflected by the formation of a lumen indicative of a normal secretory activity. Nevertheless, we further examined the presence of GATA4 by immunohistochemistry (Fig. S2A). In mature adult testes, GATA4 is primarily present in Sertoli cells. The results showed a typical Sertoli cell staining as well as the presence of SCO tubules with an apparently normal quantity of Sertoli cells in mutant testes. Similarly the expression of several other classical markers of Sertoli cell function such as Inha (α-inhibin), Gata1, kitl (Steel), and Trf (transferrin), was even higher in mutant testes at the mRNA level (Fig. S2B). Thus, the markers suggest that Sertoli cells are present and normally differentiated in mutant animals. Taken together, these observations suggest that the testicular defects observed in *hsp90α^gt/gt^* mutants are unlikely to result from a dysfunction of Sertoli cells or a defect in responding to androgens.

### Pachytene arrest in *hsp90α* mutant germ cells

To determine more precisely at which meiotic stage spermatogenesis gets disrupted in *hsp90α^gt/gt^* seminiferous epithelium, we examined the expression of spermatogenic markers at P44. Haploid-specific transcripts were absent, confirming that germ cell differentiation beyond the meiotic division was halted (Fig. 4B). Similarly, Ccna1 (Cyclin A1), Sprm1 and Cdc25c transcripts, which should appear in late pachytene spermatocytes, were drastically reduced in *hsp90α^gt/gt^* testes (Fig. 4C). The level of expression of Hoxa4, which is expressed from mid-pachytene spermatocytes to when round spermatids are formed [22], was also significantly reduced (Fig. 4C). Overall, these findings demonstrate a germ-cell specific blockade in meiotic progression at the mid to late pachytene transition.

**Figure 5:**
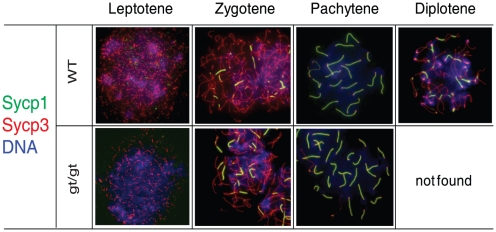
Chromosome spreads of meiotic cells. Absence of the diplotene stage in *hsp90α^gt/gt^* mutant germ cells of animals at P28. Staining of Sycp1 (in green) and Sycp3 (in red) reveals normal chromosome synapsis in *hsp90α^gt/g^* ^t^ (gt/gt) mutant germ cells until pachytene. There is a complete absence of diplotene spreads in *hsp90α^gt/gt^* mutants. Blue, DAPI staining of DNA.

Meiosis and progression through it are tightly controlled processes. It is during the meiotic prophase that homologous chromosomes recombine. Meiotic recombination involves the programmed induction of DNA double-stranded breaks (DSBs) at the beginning of prophase. These DSBs are then repaired by interaction with the homologues during the zygotene and pachytene stages. In coordination with DSB repair, homologous chromosome axes become tightly associated through a proteinaceous structure called the synaptonemal complex (SC). During pachytene, homologs are fully synapsed along their length, DSB repair proceeds and is completed at the end of pachytene leading to crossing-overs [23]. When meiosis progresses into diplotene, the SC disassembles and homologous chromosomes remain connected at the sites of crossing-over. Defects in recombination or synapsis in spermatocytes lead to a meiotic arrest in response to the pachytene checkpoint [24]. Therefore, to investigate the meiotic defect in more detail, we prepared surface spread chromosomes from *hsp90α^gt/gt^* spermatocytes. Staining for the lateral and central SC components Sycp3 and Sycp1, respectively, revealed a normal chromosome integrity and synapsis of homologues in mutant spermatocytes in prophase I (Fig. 5). Likewise, the formation of DSBs as revealed by γH2AX staining as well as ongoing repair highlighted by DMC1 staining were normal and indistinguishable from wild-type (Fig. S3). There was also no change in the successful formation of crossing-overs and hence successful homologous recombination as shown by MLH1 staining. Strikingly, we did not find any chromosome spreads indicative of stages beyond pachytene in *hsp90α^gt/gt^* samples (Fig. 5 and S3). These results demonstrate that Hsp90α is neither required for meiotic recombination nor SC formation. In contrast, Hsp90α seems to be indispensable for SC disassembly and/or progression through late pachytene or the pachytene checkpoint.

### The presence of Hsp90α is crucial during the first wave of spermatogenesis

Unlike the permanent disruption of Hsp90α expression in gene trap mutant line 1 (data not shown), we were surprised to find a somewhat different situation with line 2. A detailed analysis of the expression of Hsp90α in the testes of gene trap mutant line 2 over time revealed that the expression of Hsp90α, albeit initially strongly reduced, resumes around P45 (Fig. S1B). The Hsp90α protein levels progressively increase until, at 8 months, they reach the levels found in wild-type animals in all tissues analysed (Fig. S1B and S1C). Despite the fact that the Hsp90α expression was not permanently disrupted in this mutant, the adult males remained infertile (data not shown). These data support a crucial role of Hsp90α in the mouse before puberty when the first wave of spermatogenesis takes place.

### Exploring the mechanism

To explore the molecular mechanism responsible for the *hsp90α* mutant phenotype, we wanted to look at changes of potential Hsp90 target proteins that are known to play a role in spermatogenesis, particularly relating to the pachytene arrest. One obvious difficulty of this type of analysis is that the cell populations are altered in the testes of *hsp90α^gt/gt^* mutant animals with ultimately a very severe depletion of germ cells relative to somatic cells. Although this must be taken into account in interpreting observed changes, it should be emphasized that the depletion is progressive and that only the late stages of spermatogenesis are missing early on, for example at P21. With this caveat in mind, we compared the levels of various proteins in protein extracts from the testes of *hsp90α^gt/gt^* (mutant line 1) and wild-type animals from the age of P12 (onset of pachytene) to P44 (completion of the first spermatogenic wave) by immunoblotting.

**Figure 6:**
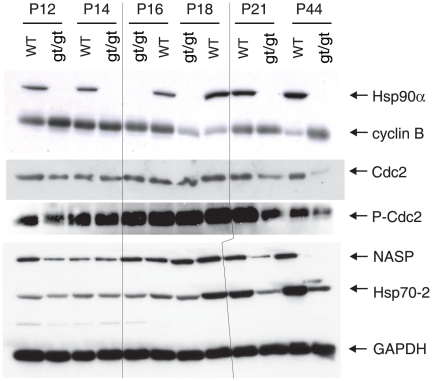
Immunoblot analysis of a panel of Hsp90- and Hsp70-related proteins. The results shown are from a representative analysis of testis extracts from wild-type and mutant animals from P12 until completion of the first wave of spermatogenesis at P44. GAPDH serves as loading control. Note that the order of the sample pairs is reversed for the P16 and P18 samples at the center of the blots (area highlighted by hairlines).

Hsp70-2 ( =  Hspa2) is a testis-specific isoform of the molecular chaperone Hsp70, which is associated with SCs [25]. In Hsp70-2 knock-out mice, the transition of spermatocytes from prophase I to metaphase I fails to occur as well [26]. Moreover, it had been shown that an unspecified Hsp70 isoform coprecipitates with both Hsp90 isoforms in murine testis extracts [27]. We therefore investigated the levels of Hsp70-2 in testicular extracts from *hsp90α^gt/gt^* and wild-type mice. Already somewhat reduced at P16, Hsp70-2 protein levels were further diminished to only 30% at P44 in mutants relative to wild-type mice (Fig. 6).

Cyclin B and Cdc2 ( =  Cdk1) form the maturation/M-phase promoting factor (MPF), which is essential for meiotic progression [28]. The spermatogenesis arrest in cyclin A1 or Hsp70-2 knock-out mice appears to be the result of a failure to activate Cdc2 by dephosphorylation [29], [30]. Since both cyclin B [31] and Cdc2 [32] are known Hsp90 clients, we examined the protein levels of cyclin B and Cdc2, and the phosphorylation status of Cdc2. In protein extracts of *hsp90α* ^gt/gt^ mouse testes, the levels of total Cdc2 were already decreased at P21. At P44, an increase of cyclin B with a concomitant reduction of Cdc2 was observed compared to the wild-type controls (Fig. 6, panels 2 and 3). The levels of the inactive Tyr15-phosphorylated form of Cdc2 in *hsp90α^gt/gt^* as compared to the wild-type animals at the latest stages of the spermatogenic progression were lower, but not as pronounced as for the de-phosphorylated form (Fig. 6, panel 4). This indicates an even more pronounced relative reduction of the active (dephosphorylated) form, potentially contributing to the pachytene arrest.

Nuclear autoantigenic sperm protein (NASP) binds and transports the testis-specific linker histone H1t to the nucleus. Although the H1t knock-out in the mouse did not affect spermatogenesis [33], NASP was demonstrated to be involved with cell cycle progression in tissue culture cells [34], probably through an interaction with the Cdc2/cyclin B and Hsp70-2 complex [35]. Moreover, it was suggested to be specifically associated with Hsp90α and to stimulate its ATPase activity [36]. The immunoblots of the testes of *hsp90α^gt/gt^* and wild-type animals showed a dramatic reduction in NASP levels in mutant animals, beginning at age P18 (Fig. 6, panel 5). These findings are compatible with an involvement of Hsp90α in NASP activation and/or stabilisation.

### An Hsp90 inhibitor disrupts testicular development

**Figure 7:**
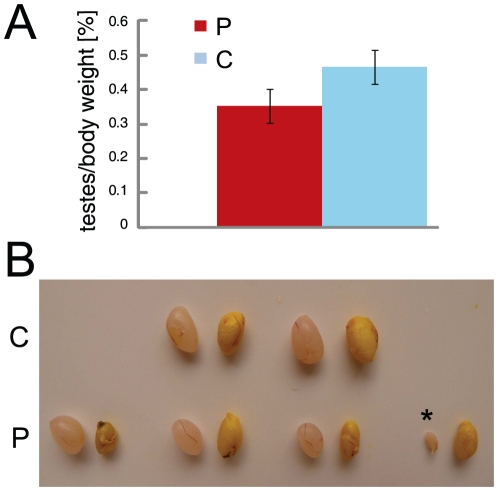
The Hsp90 inhibitor pochoxime affects testis growth. (A) Reduction of testis weight upon intraperitoneal administration of pochoxime A or vehicle. n = 6 for treated, n = 5 for control animals; p = 0.01. (B) Exterior morphology of mouse testes at P25 after administration of pochoxime A or vehicle. Testis size is reduced, in some cases atrophic (asterix). C, vehicle; P, pochoxime A.

Hsp90 is a promising drug target for anticancer therapies and several drug candidates are already in clinical trials [37]. In light of our genetic data, we decided to determine whether an Hsp90 inhibitor could induce a pharmacological phenocopy. Although geldanamycin and its derivatives have been extensively characterized and widely used, including in clinical trials [37], these do unfortunately not pass the blood-brain and blood-testis barriers [38]. Therefore, we resorted to pochoxime A, a new radicicol derivative with a promising cellular efficacy and lower toxicity compared to geldanamycin and radicicol [39]–[41]. Note that pochoxime, like all other known Hsp90 inhibitors, does not discriminate between the Hsp90α and Hsp90β isoforms. In a pilot experiment, a single intraperitoneal injection of a high dose of pochoxime (100 mg/kg of body weight) into an adult male mouse (4 months old) did not result in any obvious abnormalities three weeks later (n = 2, data not shown). We then decided to assess the effects of the continuous presence of the inhibitor in pubertal males throughout the first wave of spermatogenesis from P12 to P25. Whereas the animals did not present any internal organ abnormalities, the overall weight of the testis of the treated group was around 25% smaller than that of the control group, and some of the testes of the treated animals even became atrophic (Fig. 7). In the latter, histological examination revealed a complete disruption of the organization of the seminiferous epithelium, apparently different from the specific pachytene arrest observed in Hsp90α mutant animals (data not shown). Although the drug-induced phenotype is not identical to that of the genetic disruption of Hsp90α, it is testis-specific. The differences between the genetic and drug-induced phenotypes might stem from the fact that in the mutated animals, Hsp90α is absent during the complete process of spermatogenesis, while pochoxime A was injected only during a short interval around the end of the pachytene stage in the mouse. The fact that the drug inhibits both Hsp90 isoforms might also contribute to these differences.

## Discussion

Until now, the specific functions of Hsp90α have not been investigated genetically at the organismic level in mammals. The results of our work have shown an apparently exclusive requirement for this particular isoform for spermatogenesis. It appears that Hsp90α must be present at least during the first wave of spermatogenesis, and its absence cannot be compensated by Hsp90β and has long lasting consequences even if Hsp90α expression resumes later. In the absence of Hsp90α, meiosis arrests very specifically towards the end of the pachytene stage, homologous chromosomes fail to disassemble and normal diplotene spermatocytes are totally absent. Subsequently, there is a progressive loss of germ cells in meiosis resulting in empty seminiferous tubules and a reduced testicular size. Based on these observations and on previous knowledge from the literature, we would like to suggest that the molecular chaperone Hsp90α, possibly through an interplay with Hsp70-2, is involved directly or indirectly in the disassembly of the SC.

We were surprised to find that the only apparent phenotype caused by the absence of Hsp90α is an azoospermia due to a very specific meiotic arrest in pachytene. Even though Hsp90β is quite ubiquitously present throughout the body, one might have expected additional defects based on the emerging notion that there may be isoform-specific functions. Investigating some of these, such as the role of Hsp90α in antigen presentation and in cancer metastasis, would require specific tests and challenges that we have yet to set up. Considering the relatively high levels of expression of Hsp90α in brain and testis, it is gratifying that at least one of these organs showed an obvious phenotype.

Both Hsp90 isoforms are present in the mammalian testis. Contrary to Hsp90β, which is mainly expressed in Sertoli cells in the mouse, Hsp90α expression is more specific to primordial and mature germ cells [42], [43], although some expression in Sertoli cells is also seen [27]. In this complex organ, Hsp90β, despite being expressed, is clearly not able to complement for the loss of Hsp90α. Its levels, which apparently are not compensated in *hsp90α* mutant mice, might be too limiting in germ cells. Another surprising observation is the absence of a comparable phenotype in *hsp90α* mutant females (data not shown). In view of the fact that the knowledge about the regulation of the cell cycle and apoptosis in mammalian meiosis is still rather limited [44]–[46], this remains difficult to interpret. A previous publication had speculated that the defect in female meiosis of an Hsf1 mouse knock-out could be due to severely reduced Hsf1-controlled levels of Hsp90α [10]. While this may have contributed, the absence of Hsf1 is likely to have perturbed the expression of a whole panel of factors and the Hsp90 inhibitor, which was shown to mimic some of the defects of the mutation in an *in vitro* assay for oocyte maturation, would have inhibited Hsp90α and Hsp90β alike. The role of Hsp90 in oogenesis is nevertheless worth further investigations as a mutation in the single gene encoding the cytosolic Hsp90 gene in the nematode *Caenorhabditis elegans* was reported to result in an arrest at the prophase/metaphase boundary [47]. Unfortunately, the impact on spermatogenesis in this organism was not evaluated.

We used a candidate gene/protein approach to try to uncover the molecular mechanisms underlying the phenotype of the *hsp90α* mutation. On the one hand, we compared our phenotype to that of mutations in other genes that yield a spermatogenesis defect in the mouse and that encode Hsp90 clients and more generally any Hsp90- or Hsp70-related proteins. On the other hand, we determined whether the levels of certain candidate proteins were affected by the mutation since degradation is a common hallmark of Hsp90 clients upon Hsp90 inhibition. Considering the global role of Hsp90 and the complexity of its interactome, speculating about possible relevant targets is not trivial. A confounding difficulty stems from the fact that the relative contributions of different cell populations in the testis are altered in mutant animals. Nevertheless, this analysis suggests that multiple but perhaps interlinked pathways may be affected resulting specifically in a pachytene arrest and overall disruption of spermatogenesis.

No reported deletion of a gene encoding an Hsp90 client or cochaperone with a spermatogenesis defect in the mouse gives exactly the same phenotype [45], [48]. That of the *hsp70-2* knock-out perhaps most closely resembles the phenotype of the *hsp90α* mutation. In this case, spermatogenesis also proceeds until the final pachytene substage, the chromosomes align but fail to disassemble and normal diplotene spermatocytes are absent [49]. Since Hsp70-2 binds SCs and has been proposed to be involved in orchestrating the crucial cell cycle transition that is relevant to our phenotype [35], several additional players were worth considering. In keeping with the hypothesis, Cdc2 levels remain unchanged in *hsp70-2* mutant testes but Cdc2 kinase activity fails to be acquired [30]. Unfortunately, the mouse deletion mutants of the genes for NASP, cyclin B1, and Cdc2 ( =  Cdk1) are not informative since they are embryonically lethal [50]–[52]. The knock-out of the gene for the testis-specific cyclin isoform A1 in turn does display a spermatogenesis defect, but meiosis is arrested at diplotene [53], that is later than in our *hsp90α* mutant. In the case of the *cdk2* knock-out, there is a spermatogenesis defect associated with a pachytene arrest, but it is associated with aberrant chromosome pairing [54], which we have not observed. Surprisingly, the knock-out of the mouse gene for the testis-specific histone H1t does not affect spermatogenesis [33].

Coincident with the observation that the lack of at least some of these Hsp90/Hsp70-2-related proteins leads to a spermatogenesis defect in the mouse, we see changes in the abundance of some of these proteins. Specifically, the levels of NASP, Hsp70-2 and Cdc2, both total and in its dephosphorylated active form, are reduced. For the Hsp90 client Cdc2, this could be expected, in particular because the mere reduction of Hsp90α expression had been demonstrated to cause an instability of Cdc2 and a block of cell cycle progression in tissue culture cells [55]. For NASP and Hsp70-2, which could rather be classified as Hsp90 coregulators or partners, this would be rather unexpected. Whether there is indeed an interaction between Hsp90α and Hsp70-2, as suggested by the earlier observation of a Hsp90-Hsp70 complex in testis extracts [27], and whether Hsp70-2 is destabilized in the absence of Hsp90α, remains to be seen. As pointed out before, we cannot easily standardize these protein levels to the remaining amounts of spermatocytes relative to the other cell types in testis. However, we find these changes as early as P21, when the depletion of germ line cells is still in its early stages. We therefore speculate that the reduced levels of some or all of these regulatory proteins impair the formation of the H1t/NASP/Hsp-70/Cdc2 complex and the transition to the activation of the Cdc2/cyclin B complex (MPF) for progression beyond pachytene. An alternative, albeit not mutually exclusive, hypothesis builds on the similarity of the *hsp70-2* and *hsp90α* mutant phenotypes and on the observation that Hsp70-2 localizes to SCs [49]. Unless the effects on the levels of the afore-mentioned proteins are coincidental, it is tempting to speculate that the molecular chaperones Hsp70-2 and Hsp90α may be involved in the disassembly of SCs.

Our finding that an Hsp90 inhibitor can phenocopy the *hsp90α* mutation, at least when applied during puberty, may have implications for the future clinical use of such drugs. Several Hsp90 inhibitors are currently in clinical trials as anti-cancer drugs [37] and may eventually also be explored for even more long-term treatments, for example against neurodegenerative diseases. It is presently not known whether such a therapy would have an impact on male fertility. In this context, it is noteworthy that we achieved the pharmacological modulation of male fertility in mice at ten-fold lower doses than those commonly applied for tumor inhibition [39]. Considering the impact of a genetic disruption of the *Hsp90α* gene or of the pharmacological treatment with a drug that is not Hsp90 isoform-specific, the potential side effects of the current generation of drugs and, perhaps some time in the future, of isoform-selective ones need to be more carefully evaluated. Unlike the effects of an application during puberty, as in our experiments with mice, specific Hsp90 inhibitors given to human adults may well prove to be innocuous for fertility. And yet, caution is warranted as further emphasized by the finding that gamendazole, which has been advocated as a potential oral and nonhormonal single dose male contraceptive, apart from other protein(s) also binds Hsp90 albeit with a distinct pharmacological profile than inhibitors targeting the N-terminal nucleotide binding pocket [56].

## Materials and Methods

### Ethics statement

Animal husbandry and all animal experimentation was carried out in compliance with Swiss laws. Approval was obtained from the University of Geneva “animal experimentation ethics committee” (protocol number 10–11) and formal authorisations (numbers 31.1.1027/3263/1 and 31.1.1027/3576/2) were obtained from the State and Federal authorities.

### Generation of mutant mouse lines and genotyping

The 129P2 ES cell clone XE444 with the gene trap vector pGT1Lxf in the *Hsp90aa1* gene was obtained from Baygenomics, now part of the International Gene Trap Consortium (http://www.genetrap.org). The mouse line *Hsp90aa1^Gt(XE444)Byg^* was established by injection of XE444 cells into C57BL/6 blastocysts and is herein referred to as mutant mouse line 1 or *hsp90α^gt/gt^*. After germ line transmission was obtained, mice were maintained by breeding with C57BL/6. The second mouse line, *Hsp90aa1^Gt(S17-2G1)Sor^* (herein referred to as mutant mouse line 2) was generated from the 129S4/SvJae ES cell clone S17-2G1 containing the gene trap vector ROSAFARY in the *Hsp90aa1* gene. Chimeras for this line were obtained from the Mutant Mouse Regional Resource Centers at the University of California, Davis (http://www.mmrrc.org). Genotyping by PCR was done with DNA isolated from tail or ear biopsies using the Direct PCR Lysis Reagent (Viagen Biotech). PCR primers were directed to the integrated gene trap sequence and the flanking parts of the *Hsp90aa1* intron 10 and exon 11 for line 1, and sequences around the insertion site of the ROSAFARY gene trap vector in intron 1 of the *Hsp90aa1* gene for line 2. Primer details are given in Table S1.

### Immunoblotting

Protein extracts were prepared by homogenisation of mouse organs in protein isolation buffer (10 mM Tris-HCl pH 8, 1% Triton-X100, 2 mM EDTA, 10% glycerol, 137 mM NaCl, phosphatase and proteinase inhibitor cocktails) with an Amaxa tissue homogenizer, sonicated, and resolved by SDS-polyacrylamide gel electrophoresis. For immunoblotting, the following antibodies were used: the Hsp90α-specific polyclonal antibody PA3-013 (Affinity BioReagents), the monoclonal Hsp90β-specific antibody H90-10 (a kind gift of Dr. David Toft), the rabbit polyclonal antisera 9112 and 9111 against Cdc2 and phospho-Cdc2 (Cell Signalling), respectively, the NASP-specific polyclonal antibody 72494 (Abcam), the monoclonal antibody HPA000798 against Hsp70-2 (Atlas Antibodies AB), the monoclonal antibody GNS11 against cyclin B (Neomarkers), and the monoclonal anti-GAPDH antibody 6C5 (Abcam). In some cases, immunoblots were quantitated by acquiring the chemiluminescent signal with a Syngene GeneGnome.

### Histological examination and immunohistochemistry

Freshly dissected testes were fixed in Bouin's solution for hematoxylin and eosin staining and in 4% paraformaldehyde for immunostaining, washed in phosphate-buffered saline (PBS) and embedded in paraffin. Eight 5 µm sections per adult testis, taken at different levels, were selected for investigating the size of the seminiferous tubules and to assess the different stages of spermatogenesis. For immunohistochemistry, the sections were stained with a 50-fold dilution of the polyclonal GATA4 antibody sc-9053 (Santa Cruz Biotechnology) after standard antigen retrieval. Images were taken with a Zeiss AxioCam microscope.

### Sperm counts

To determine the epididymal sperm counts, the caudal epididymides and deferent ducts were surgically removed from adult mice, washed with PBS, cut into pieces with scissors, and carefully squeezed with forceps in order to release the sperm content into 250 µl of pre-warmed M2 medium. After eosin-negrosin staining, the total epididymal sperm counts were assessed using a Neubauer hemocytometer.

### Flow cytometry

Flow cytometric analyses were done essentially as previously described [57]. Briefly, testes from P44 wild-type and *hsp90α^gt/gt^* mice (n = 3 per group) were dissected and decapsulated to release the tubules, which were then incubated in 0.25 mg/ml collagenase type IV (Sigma) at 37°C for up to 5 min with rapid agitation. Dispersed tubules were allowed to settle and washed twice to remove peritubular cells. Washed tubules were then incubated with 0.5% Trypsin EDTA (Gibco) and 1 µg/ml DNase RQ1 (Promega) at 37°C for 5 min with agitation. Trypsin digestion was terminated by adding DMEM with 10% FCS. The suspension was washed and disaggregated into a single-cell suspension by trituration using a flame-polished Pasteur pipette and filtered through a 50 µm cell strainer. For DNA content and nuclear analysis, germ cells were rinsed and resuspended in 1 ml of cold propidium iodide (PI) staining solution (10 mM Tris-HCl pH 8.0, 1 mM NaCl, 0.1% Nonidet-P40, 50 µg/ml PI, 10 µg/ml RNaseA), vortexed for 2–3 sec and incubated on ice for 10 min to lyse the plasma membrane and stain nuclear DNA. DNA content was assessed on a FacsCalibur II (Becton-Dickinson) equipped with the CELL Quest software.

### Real-time quantitative RT-PCR

This was performed essentially as previously described [58]. Total RNA from the testes of P44 mice (5 wild-type and 7 mutants) was extracted with the Trizol reagent. Subsequently, RNA preparations were treated with DNAse I and re-purified with the RNeasy minicolumn elution kit from QIAGEN according to the manufacturer's instructions. RNA integrity and quantity were assessed with an Agilent 2100 bioanalyzer, using RNA 6000 nanochips. Total RNA samples were reverse transcribed with the Superscript III reverse transcriptase (Invitrogen) according to the manufacturer's protocol. One-twentieth cDNA template was used as template for each PCR reaction. cDNA was PCR amplified in a 7900HT Sequence Detection Systems (Applied Biosystems) using the Power SYBR Green PCR master mix (Applied Biosystems). Raw threshold-cycle (Ct) values were obtained with the Sequence Detection Systems 2.0 software (Applied Biosystems). Relative quantities (RQs) were calculated with the formula RQ = E−Ct, using efficiencies calculated for each run with the Data Analysis for Real-Time PCR (DART-PCR) algorithm, as described [59]. A mean quantity was calculated from triplicate PCR reactions for each sample, and this quantity was normalized to two similarly measured values of normalization genes (glyceraldehyde-3-phosphate dehydrogenase and β-actin). Normalized quantities were averaged for seven wild-type and five mutant animal samples. The normalized relative quantity of the wild-type was arbitrarily given a value of 100% and the % change was calculated for the mutant animals. They were expressed +/−standard error. Details on primers are given in Table S1.

### Meiotic chromosome spreads

Spreads were prepared by the dry-down technique as previously described [60]. Briefly, testes were dissected, the albuginea layer removed, tubules cut into small pieces and dissociated with a pipet in PBS. Cells were ruptured by adding equal amounts of hypotonic buffer (30 mM Tris-HCl pH 8.2, 50 mM sucrose, 17 mM sodium citrate, 5 mM EDTA, 0.5 mM DTT, protease inhibitors) and mixed 1∶2 with 100 mM sucrose just before spreading on the slides. The slides were dipped in the paraformaldehyde solution, incubated in a humid chamber for 1 h and then dried for 30 min. Slides were washed 2×1 min with 0.08% Photo-flo (Kodak). Immunostaining of spermatocyte spreads was carried out as described [61], using a milk-based blocking buffer (5% milk, 5% donkey serum in 1xPBS). Antibodies used were: monoclonal antibodies against Sycp1 (a gift from C. Heyting), phospho-H2A.X (Upstate) and MLH1 (Pharmingen), a guinea pig antiserum against Sycp3 and a rabbit antiserum against DMC1 (Santa Cruz Biotechnology) at 500-, 25'000-, 50-, 500-, and 200-fold dilutions, respectively. All incubations with primary antibodies were carried out overnight at room temperature. Secondary antibodies were goat anti-guinea pig Alexa Fluor 488 (Molecular probes), donkey Cy5-conjugated antimouse and Cy3-conjugated anti-rabbit antibodies. Incubations with secondary antibodies were carried out at 37°C for 90 min. Nuclei were stained with DAPI (2 mg/ml) during the final washing step. Digital images were obtained by using a cooled CCD camera, Coolsnap HQ (Photometrics), coupled to a Leica DMRA2 microscope using the same exposure time for all aquisitions. Each colour signal was acquired as a black-and-white image using appropriate filter sets and was merged with Photoshop Imaging software using the same entry levels for each histone modification in order to be able to compare the staining intensity at different stages of meiosis.

### Pochoxime inhibitor study

Wild-type B6CBA F1 males were injected intraperitoneally every day from P12 to P25 with 10 mg of pochoxime A per kg of body weight. The inhibitor was dissolved in DMSO to 100 µg/µl and stored at −20°C. A working dilution of 1 µg/µl was prepared by diluting the pochoxime A stock solution first with Tween-20 and then further with a 0.9% NaCl solution at a volume ratio of 1∶0.5∶8.5. Wild-type controls were injected with the same solvent without pochoxime A. Animals were sacrificed for analysis on P25.

## Supporting Information

Figure S1 Functional *hsp90*α gene disruption mouse line 2. (A) Schematic representation of the disruption of the mouse *Hsp90aa1* gene by insertion of a gene trap (GT) into the first intron. Open and black boxes indicate non-coding and coding exons, respectively. The gene trap consists of a splice acceptor (SA), a β-galactosidase-neomycin resistance fusion (βGeo) and an SV40 polyadenylation site. (B) and (C) Immunoblots showing the expression of Hsp90α and Hsp90β in wild-type (+/+) and mutant (−/−) mice; in testes over time after birth for panel B and in different tissues at 8 months in panel C. (D) Time course analysis of testis weight in WT and mutant (gt/gt) animals. (E) Histological sections of testes of of wild-type (WT) and mutant (gt/gt) mice at P70. Two different magnifications are shown.
(TIF)

Figure S2 Sertoli cell function appears normal. (A) Immunohistochemical staining for the Sertoli marker GATA4 in 4 months old testes of wild-type and *hsp90α^gt/gt^* mutant animals. Asterix indicates a SCO tubule. Blue, DAPI staining; green, GATA4 staining. (B) Quantitative RT-PCR analysis of other Sertoli cell-specific markers at P44.
(TIF)

Figure S3 DSB repair processes and homologous recombination proceed normally in *hsp90α^gt/gt^* spermatocytes. Staining for γH2AX (green) reveals the formation of DSBs, first detected at leptotene, DMC1 (green) at sites of DSB repair and MLH1 (green) at sites of crossing over. Chromosome axes are stained with Sycp3 (red) and DNA with DAPI (blue). Samples are from animals at P28.
(TIF)

Table S1 Oligonucleotides used for genotyping and quantitative RT-PCR.
(DOC)
