# Supplementary material for: The Molecular Chaperone Hsp90α Is Required for Meiotic Progression of Spermatocytes beyond Pachytene in the Mouse
Source: PLoS One. 2010 Dec 31;5(12):e15770. doi: 10.1371/journal.pone.0015770 (PMC3013136; doi:10.1371/journal.pone.0015770)
Supplement: Table S1 — Oligonucleotides used for genotyping and quantitative RT-PCR. (DOC) [file pone.0015770.s004.doc]

Table S1A: Oligonucleotides used for genotyping

| **Mutant mouse** | **Target** | **Oligonucleotide** |
| --- | --- | --- |
| Line 1 | *Hsp90aa1* intron 10  *Hsp90aa1* exon 11  Gene trap vector pGT1Lxf | GCTGTTATGGAAGCCTCAGC  AGGGTTGTTCTCGGGACTTT  CACTCCAACCTCCGCAAACTC |
| Line 2 | *Hsp90aa1*  intron 1  *Hsp90aa1*  intron 1  Gene trap vector ROSAFARY | CTTCAGAACACACTCCAGCG  TCTAAGGCTTCTTTTCCCCG  AACCCTGGACTACTGCGCCC |

Table S1B: Oligonucleotides used for quantitative RT-PCR

| **mRNA^1^** | **Forward** | **Reverse** |
| --- | --- | --- |
| Actb (β-actin) | CTAAGGCCAACCGTGAAAAGAT | CACAGCCTGGATGGCTACGT |
| Amh (MIS) | TAGTCCTACATCTGGCTGAAGTGATATG | CCAGGTGGAGGCTCTTGGA |
| Ccna1 (Cyclin A1) | CACTTCCTGCTGGATTTCAAC | CGATGAATCTCCTCTGCATAC |
| Cdc25c (Cdc25) | ATTGCCGCTATCCGTATGAGTAC | TCCTTCTGACTGTGCAGGTTTAAG |
| Gapdh | TCCATGACAACTTTGGCATTG | CAGTCTTCTGGGTGGCAGTGA |
| Gapdhs (spermatogenic GAPDH) | TAGAGCCAGACAAGTTTGAAG | TGCGTCCAAATCCATTGATAC |
| Gata1 | TGTGTGAACTGTGGAGCAACGGC | AAATAGAGGCCGCAGGCATTGCA |
| Hoxa4 | TGTACCCCTGGATGAAGAAGATC | GCTTAGGTTCGCCTCCGTTA |
| Inha (α-inhibin) | GCACAGGACCTCTGAAC | TGGGATGGCCGGAATAC |
| Insl3 | CCTGGCTATGTCATTGCAACA | TGGTCCTTGCTTACTGCGATCT |
| Kitl (Steel) | TGTGCTCTCTTCAACATTAGG | TTACTGCTACTGCTGTCATTC |
| Pou5f1 (Oct4) | CAATGAGAACCTTCAGGAGATATGC | TCAATGCTAGTTCGCTTTCTCTTC |
| Prm1 (Protamine 1) | GCCGCTCATACACCATAAGG | CAAGATGTGGCGAGATGC |
| Prm2 (Protamine 2) | GCAGAAGATCCCGAAGGAG | CTCCAGGCAGAATGGACAG |
| Sprm1 | CTAGCCAAGGAACTGAGAC | GCTGAGAACCTTCCCAAAC |
| Star | ACATATGCGGAATATGAAAGGATTAA | CCCCAGTGCTCTCCAGTTGA |
| Tnp1 | GATGCAAGTCGCAATTACC | CCCGTGTTGTTTGAAGACC |
| Tnp2 | GACACTCACCTGCAAGAC | ATCTTCGCCCTGAGCTAC |
| Trf (Transferrin) | ATCTGGGAGATTCTCAAAGTG | AGTGTGGCAGGACTTCTTGCC |

^1^ Official names are used, but in some cases, a more commonly known name is given in parenthesis.
